# A Novel Polyacrylamide Film-Forming Agent for Maintaining Wellbore Stability

**DOI:** 10.3390/molecules30193877

**Published:** 2025-09-25

**Authors:** Guoyan Ma, Wenjing Wei, Yanzhe Yang, Chao Hao, Yaru Zhang, Guoqiang Xu

**Affiliations:** 1 College of Chemistry and Chemical Engineering, Xi’an Shiyou University, Xi’an 710065, China; wwj310187631@163.com (W.W.); yanzheyang2025@163.com (Y.Y.); zhangyaru_169@163.com (Y.Z.); 17638732255@163.com (G.X.); 2 CCDC Drilling & Production Engineering Technology Research Institute, Xi’an 710018, China; kenny1989526@cnpc.com.cn

**Keywords:** polyacrylamide film-forming agent, copolymerization reaction, microscopic analysis, wellbore inhibitor, moldability test

## Abstract

A polyacrylamide-based film-forming agent was synthesized via free-radical copolymerization. FT-IR spectroscopy confirmed complete monomer conversion with no detectable residual unsaturation. Systematic variation of acrylamide (AM), vinyl acetate (VAc) and cellulose content revealed that an AM mass fraction of 3.7 wt%, a VAc:AM molar ratio of 1:3 and a cellulose content of 1.6 wt% yielded an emulsion of maximal colloidal stability. Under these conditions, the agent formed coherent, moisture-resistant films that effectively encapsulated sodium-bentonite pellets, indicating its potential as an efficient inhibitor for maintaining well-bore stability during drilling operations.

## 1. Introduction

As global demand for energy continues to grow, the exploration and development of oil and gas resources are increasingly extending into deeper and more complex geological formations, presenting unprecedented challenges for drilling operations. Wellbore stability issues have long been a major challenge in drilling operations, particularly in collapsible shale formations, high-pressure zones, and complex geological conditions. Instability of the wellbore can lead to drilling accidents, increasing operational costs and risks [1]. The primary cause of wellbore instability is the surface hydration and permeation hydration of clay minerals in mud shale when exposed to water. The main methods to address this issue include blocking water molecule intrusion, inhibiting expansion, and using physical plugging agents to reduce intrusion. Currently, commonly used inhibitors for wellbore stability issues both domestically and internationally include silicates, potassium salts, and polymers [2]. Silicate inhibitors have the advantages of strong temperature resistance and good inhibition effects, but in actual use, they easily form precipitates with calcium and magnesium ions, potentially causing stuck pipes. Potassium salt inhibitors are cost-effective and have good compatibility, but they easily lose effectiveness at high temperatures and can severely contaminate groundwater and soil. Polyetheramine inhibitors exhibit good high-temperature resistance and salt resistance, but their high cost poses significant challenges for industrialization [3,4,5]. Acrylamide, due to its low cost, can inhibit clay hydration swelling through hydrogen bonding and electrostatic interactions.

Polyacrylamide (PAM) film-forming agents are based on acrylamide copolymers or their derivatives. They are produced by altering their molecular structure, introducing new functional groups, or undergoing composite cross-linking reactions. The final product is an emulsion that forms a continuous, uniform polymer film on the surface of the target material [6,7,8,9,10]. Polyacrylamide film-forming agents are widely used in industries such as healthcare [11,12], petroleum [13,14], chemicals [15,16], coatings [17], and water treatment [18,19,20]. Polyacrylamide film-forming agents are mainly divided into the following categories.

Nonionic polyacrylamide is a polymer generated by acrylamide monomer through free radical polymerization reaction. Its molecular chain contains only -CONH_2_ group and does not contain electronic or ionic groups [21]. However, non-ionic polyacrylic acid-based film-forming agents generally have poor thermal stability and cannot withstand the high temperatures found in wells. In addition, the film strength of this type of film-forming agent is lower than that of other types of film-forming agents. Cationic polyacrylamide film-forming agents refer to polyacrylamide polymers with cationic functional groups. These film-forming agents typically have good adhesion properties and are used as flocculants in water pollution treatment [22,23], but cationic polyacrylamide film formers exhibit limited efficacy in managing salt concentrations and are associated with considerable costs.

Anionic polyacrylamide film-forming agents generally refer to polyacrylamide molecules with anionic functional groups (such as carboxyl groups, sulfonic acid groups, or phosphate groups) introduced into their molecular chains. As a result, these film-forming agents exhibit strong hydrophilic properties, excellent film-forming performance, and good stability. They dissolve well in water and can rapidly form uniform, transparent, and flexible protective films. Anionic polyacrylamide-based film-forming agents are widely used in personal care products, cosmetics, pharmaceuticals, agriculture, coatings, petroleum and other fields. Zhang et al. [24] created a flame-retardant nano-coating using anionic polyacrylamide to prevent fire hazards in flammable polymer materials. Yang et al. [25] used water-soluble anionic polyacrylamide to improve frozen soil and reduce the occurrence of frost damage. Habibi et al. [26] prepared a flocculant for wastewater treatment using anionic polyacrylamide and polyaluminium chloride, which resulted in a corresponding reduction in cost and energy consumption compared to traditional methods. Anionic polyacrylamide has a wide range of applications. However, there are few reports on its use in oilfield extraction.

Copolymerized and graft-modified film-forming agents are a class of functional polymeric materials prepared through copolymerization of different monomers or using graft modification technology [27]. By selecting specific monomers and adjusting their ratios, the chemical structure and properties of these materials can be precisely controlled, enabling the production of functional film-forming materials with specific properties. Compared to traditional single-component polymers, copolymerized and graft-modified film-forming agents can impart special functions to film-forming materials, such as anti-soiling, hydrophilicity, hydrophobicity, and antibacterial properties, meeting the specific requirements of application industries for film-forming agent materials. Currently, these film-forming agents are widely used in industries such as coatings, adhesives, textile finishing, pharmaceutical materials, electronic component protective layers, and oil displacement. El-Hoshoudy et al. [28] prepared a hydrophobic associative copolymer of polyacrylamide to improve oil recovery efficiency. Xie et al. [29] synthesized a nano-composite polyacrylate (Poly-TALC) copolymer using butyl acrylate and triallyl isocyanurate as raw materials, which are capable of achieving downhole nano-plugging in shale formations. Abd-Elaal et al. [30] generated polyacrylamide copolymers by copolymerizing cationic acrylamide with acrylamide are used to improve oil extraction efficiency.

With the continuous growth of global energy demand, oil and gas exploration and development are moving into deeper and more complex formations. This trend brings unprecedented challenges to drilling engineering. Wellbore stability has always been a major difficulty in drilling operations. In shale formations, high-pressure zones, and complex geological conditions, unstable wellbores can easily cause drilling accidents and increase operational costs and risks.

In this study, we developed a film-forming agent based on anionic polyacrylamide through emulsion polymerization, intended to be used as a drilling fluid additive to improve wellbore stability in underground oilfield operations. The performance of this agent in inhibiting the hydration and swelling of bentonite was validated through film-forming tests. The material effectively reduces the swelling and hydration of shale, which are key factors causing wellbore instability during drilling. At the same time, the material demonstrates excellent stability, high-temperature resistance, and other superior properties, making it suitable for extreme underground oilfield conditions with high temperature and pressure, and it can effectively achieve wellbore stabilization. This study introduces this novel film-forming material as a new solution for wellbore stability issues. Potassium persulfate was used as the initiator to copolymerize acrylamide, methyl methacrylate, and vinyl acetate, resulting in a polyacrylamide-based composite emulsion (AMVA). The structure and properties of the composite emulsion were systematically analyzed using Fourier transform infrared spectroscopy (FTIR), thermogravimetric analysis (TGA), dynamic light scattering (DLS), polarized light microscopy, and film-forming performance tests. This study provides a thorough investigation into the microstructure and physicochemical properties of the film-forming material.

## 2. Results and Discussion

### 2.1. Particle Size Analysis

#### 2.1.1. Effect of AM Content on the Particle Size of AMVA

**Figure 1:**
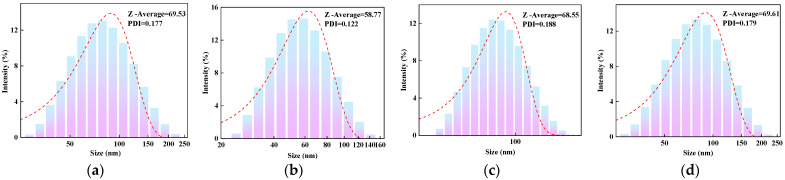
Particle size distribution curves of AMVA emulsions with different AM contents; (**a**) AMVA-1 (AM content of 1 wt%); (**b**) AMVA-2 (AM content of 3.7 wt%); (**c**) AMVA-3 (AM content of 7 wt%); (**d**) AMVA-4 (AM content of 10 wt%).

Figure 1 shows that particle sizes of AMVA-1, AMVA-2, AMVA-3 and AMVA-4 are 69.53 nm, 58.77 nm, 68.55 nm and 69.61 nm, respectively. All samples exhibit monomodal particle-size distributions. AMVA-2 possesses the smallest mean diameter, and the particle size initially decreases and then plateaus with increasing AM content. Polydispersity indices (PDI) lie between 0.10 and 0.20, confirming the narrow and uniform distribution of the emulsion particles. By precisely controlling the particle size distribution of the emulsion, a more uniform dispersion effect can be achieved, thereby enhancing the interaction with the wellbore surface. It is noteworthy that particle size analysis of film-forming agents has not been adequately emphasized in most studies [31].

#### 2.1.2. Effect of Molar Ratio of VAc to AM on the Particle Size of AMVA

**Figure 2:**
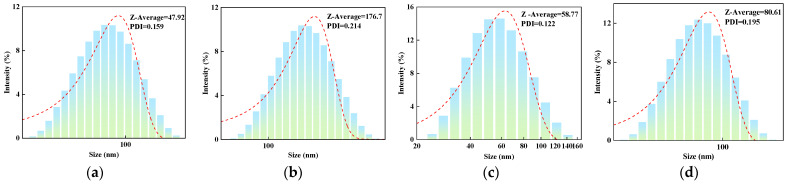
Particle size distribution curves of AMVA emulsions with different molar ratios of AM to VAc; (**a**) AMVA-5 (AM:VAc = 1:2); (**b**) AMVA-6 (AM:VAc = 1:3); (**c**) AMVA-2 (AM:VAc = 1:4); (**d**) AMVA-7 (AM:VAc = 1:5).

**Figure 3:**
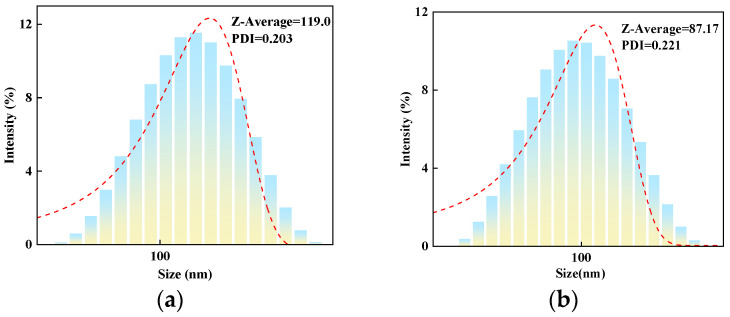
Particle size distribution curves of emulsions with different cellulose contents; (**a**) AMVA-8 (1 wt% cellulose in emulsion); (**b**) AMVA-9 (1.6 wt% cellulose in emulsion).

Figure 2 illustrates the particle size distribution of the emulsion at various molar ratios of AM to VAc, namely 1:2, 1:3, 1:4, and 1:5. As depicted in Figure 2, the corresponding particle sizes for these molar ratios are 47.92 nm, 176.7 nm, 58.77 nm, and 83.61 nm, respectively. A significant increase in particle size is observed when the molar ratio of AM to VAc is 1:3 (Figure 3b). This pronounced change is attributed to the higher vinyl acetate content, which likely induces larger particle formation during emulsion polymerization. The polydispersity index (PDI) of the emulsion ranges from 0.1 to 0.2, indicating a relatively uniform particle size distribution. Research indicates that the pore sizes of mud shale typically fall within the range of 0.1 to 10.0 µm. Based on the principles of filling and bridging, the product with a molar ratio of AM to VAc of 1:3 is more effective in plugging the pores and fractures in shale, thereby enhancing its potential for application in relevant contexts.

#### 2.1.3. Effect of Cellulose Content on the Particle Size of AMVA

The impact of cellulose content on the AMVA emulsion was examined using samples designated as AMVA-8 and AMVA-9, which contained 1 wt% and 1.6 wt% cellulose, respectively. The detailed formulations of these samples, including the specific ratios of components, are presented in Table 1.

Figure 3 presents the particle size distribution of AMVA emulsions prepared with varying cellulose contents. For the samples with cellulose contents of 1.0 wt% and 1.6 wt%, the corresponding particle sizes are 119.0 nm and 87.17 nm, respectively, both exhibiting monomodal distribution. An increase in cellulose content from 1.0 wt% to 1.6 wt% results in a reduction in particle size. However, the polydispersity index (PDI) of the AMVA emulsion exceeds 0.2, which suggests a relatively broad particle size distribution and compromised uniformity.

### 2.2. Polarizing Microscope Analysis

#### 2.2.1. Effect of Molar Ratio of VAc to AM on the Polarized Optical Microscopy Analysis of AMVA

**Figure 4:**
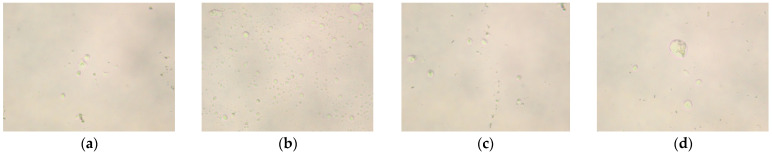
Microscopic images of emulsions with different molar ratio of VAc to AM; (**a**) AMVA-5; (**b**) AMVA-6; (**c**) AMVA-2; (**d**) AMVA-7.

**Figure 5:**
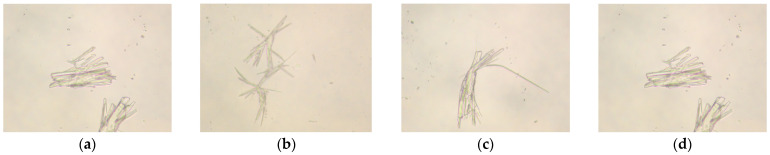
Microscopic images of bentonite aggregates after centrifugation with different molar ratio of VAc to AM; (**a**) AMVA-5; (**b**) AMVA-6; (**c**) AMVA-2; (**d**) AMVA-7.

Figure 4 displays the microscopic images of the clarified liquid subsequent to centrifugation, whereas Figure 5 presents the microscopic images of bentonite aggregates following centrifugation. In Figure 4, most of the sample particles appear spherical, with some aggregating into larger particles. Additionally, most of the particles have good dispersion, and the microscopic analysis results are consistent with the results of the particle size analysis. In Figure 5, after mixing with bentonite and centrifuging, the particles mainly look like needle-like rods. The majority of particles remain aggregated, signifying that AMVA emulsions confer pronounced stabilization to the well-bore wall.

#### 2.2.2. Effect of Cellulose Content on the Polarized Optical Microscopy Analysis of AMVA

**Figure 6:**
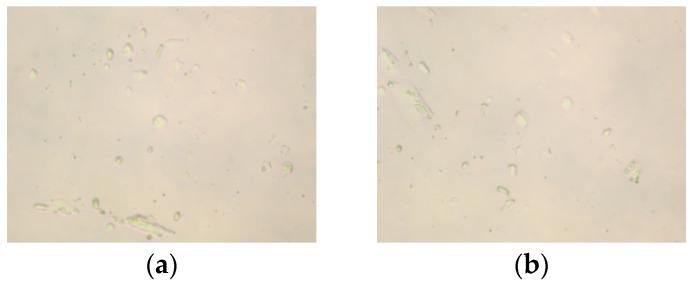
Microscopic images of AMVA emulsions with different cellulose contents; (**a**) AMVA-8; (**b**) AMVA-9.

**Figure 7:**
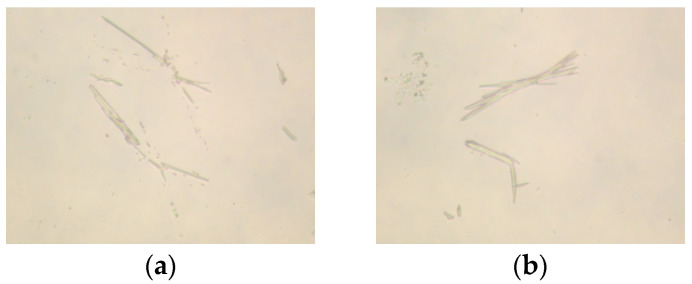
Microscopic images of bentonite aggregates after centrifugation with different cellulose contents; (**a**) AMVA-8; (**b**) AMVA-9.

Figure 6 presents optical micrographs of the post-centrifugation supernatant, whereas Figure 7 displays those of the bentonite aggregates. Figure 6 reveals that the majority of the dispersed entities appear as spherical particles; within these spheres, elongated cellulose nanofibers are distinctly discernible. Microscopic observations corroborate the particle-size data. After co-mixing with bentonite and subsequent centrifugation (Figure 6), the sample exhibits elongated, needle-like morphology. Although a minor fraction of particles remains aggregated, the overall dispersion is markedly improved, underscoring the superior borehole-stabilizing efficacy of AMVA-9.

### 2.3. FTIR of AMVA

**Figure 8:**
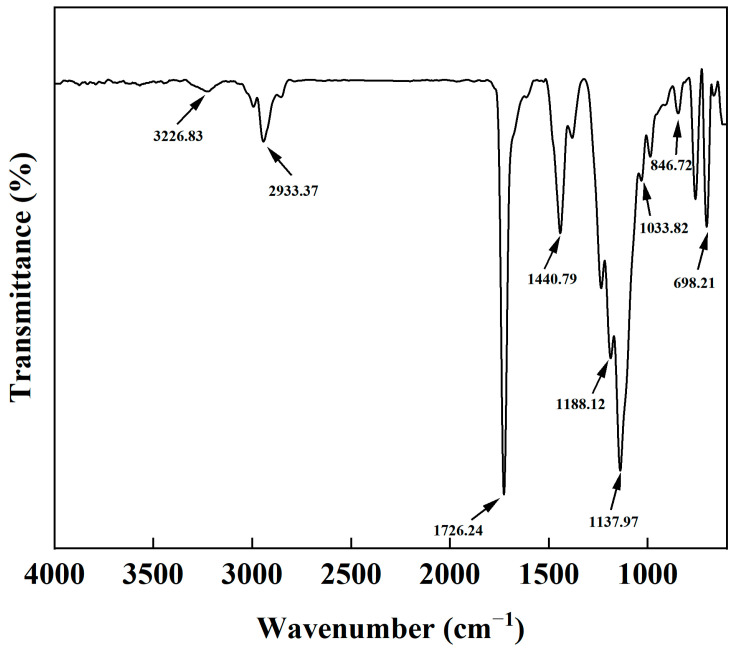
FTIR spectra of AMVA.

To identify the chemical composition of the AMVA emulsion, the molecular structure of the product was analyzed by Fourier transform infrared (FTIR) spectroscopy in the range of 4000~400 cm^−1^. Figure 8 illustrates the FTIR spectrum of the AMVA emulsion. The absorption peak at 3226.83 cm^−1^ corresponds to the stretching vibration of the amino group, and the peak at 2933.37 cm^−1^ is caused by methyl or methylene groups. The characteristic absorption peak of C=O typically appears around 1720 cm^−1^; thus, the bending vibration peak near 1726.24 cm^−1^ is identified as the C=O absorption peak. The peak located at 1440.79 cm^−1^ is due to the bending vibration of -CH_2_ groups, and the peaks at 1188.12 cm^−1^ and 1137.92 cm^−1^ are associated with the -C-O-C- vibration, confirming the successful incorporation of ester groups. The Si-O-Si stretching vibration peak is observed at 1033.82 cm^−1^, while the Si-O bending vibration peak is seen at 846.72 cm^−1^. The peak at 698.21 cm^−1^ corresponds to the planar bending vibration of the -CH bond in the phenyl ring. No characteristic C=C stretching vibration absorption peak was detected in the range of 1675–1640 cm^−1^, indicating the absence of unreacted vinyl monomer and confirming that no residual monomers are present in the polymer product. These results illustrate that the prepared AMVA emulsion contains the characteristic functional groups of the various monomers designed in its molecular structure.

### 2.4. Thermogravimetric Analysis of AMVA

**Figure 9:**
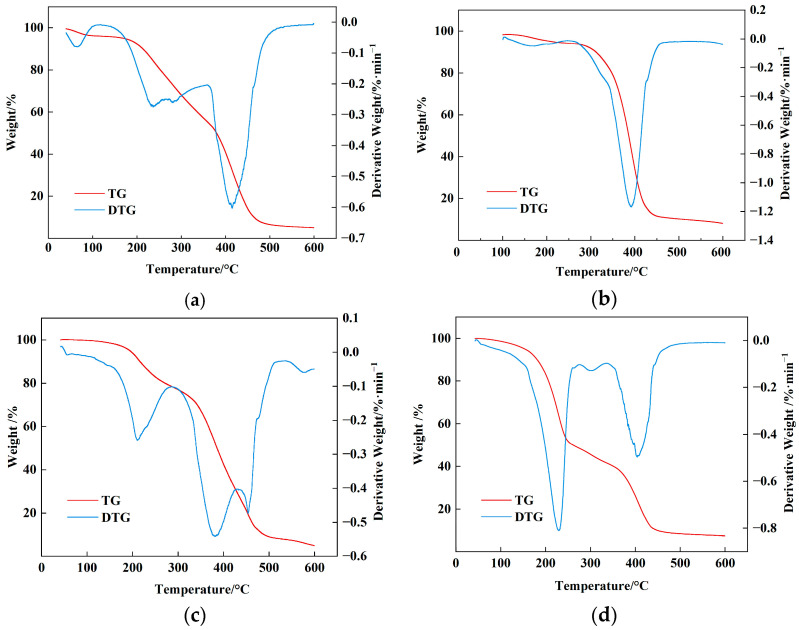
TG and DTG curves of AMVA at different AM contents; (**a**) AMVA-1 (AM content of 1.0 wt%); (**b**) AMVA-2 (AM content of 3.7 wt%); (**c**) AMVA-3(AM content of 7 wt%); (**d**) AMVA-4 (AM content of 10 wt%).

Thermogravimetric analysis (Figure 9 and Table 2) demonstrates that the principal mass-loss interval for all samples lies between 200 °C and 450 °C. The onset decomposition temperature first rises from 117 °C to a maximum of 246 °C, then declines to 120 °C; AMVA-2 exhibits the highest decomposition temperature among the series. Similarly, the half-decomposition temperature (T_1/2_) increases from 379 °C to 386 °C and subsequently decreases to 261 °C. These results confirm that an AM mass fraction of 3.7 wt% in the AMVA emulsion markedly enhances thermal stability. Sun et al. developed an organosilicon salt inhibitor named KMAA, which has an initial thermal decomposition temperature of 107 °C. Compared with the study by Sun et al., the AMVA emulsion prepared in this study shows better thermal stability [32]. This is mainly attributed to the optimized formulation of the AMVA emulsion, while the incorporation of St and MMA increases the rigidity of the polymer chains, thereby significantly enhancing the structural stability of the emulsion.

Differential thermogravimetric (DTG) analysis resolved multiple degradation events that remained obscured in the corresponding TG profile. The second mass-loss step exhibited a pronounced, positive correlation with AM concentration, confirming that scission of acrylate and methacrylate moieties dominates this stage. An initial, lower-temperature transition reflects the onset of side-chain cleavage, succeeded by a prominent DTG maximum at 370~400 °C that corresponds to backbone fragmentation. A subsequent peak at 480~500 °C is ascribed to the thermal decomposition of γ-MPS-derived organosilicon functionalities.

In general, the thermal stability of a polymer is dictated by its molecular architecture and the inherent thermal robustness of the constituent functional groups. Progressive elevation of the AM fraction raises the density of amide functionalities along the macromolecular backbone; these amide groups establish an extended hydrogen-bonding network that strengthens intermolecular cohesion and, consequently, elevates thermal endurance. Thermal analyses indicate that the AMVA emulsion reaches maximal thermostability when the AM content is precisely 3.7 wt%. Beyond this threshold, excessive intermolecular crosslinking and non-uniform chain packing emerge, disrupting the regularity of the network and precipitating a discernible decline in thermal stability.

### 2.5. Moldability Test

#### 2.5.1. Effect of Acrylamide Content on Film-Forming Properties of AMVA Emulsions

**Figure 10:**
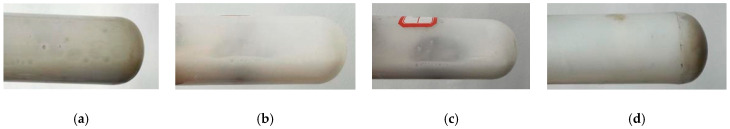
Effect of AM content on the film-forming properties of AMVA emulsions; (**a**) AMVA-1 (AM content of1 wt%); (**b**) AMVA-2 (AM content of 3.7 wt%); (**c**) AMVA-3 (AM content of 7 wt%); (**d**) AMVA-4 (AM content of 10 wt%).

At 25 °C, 40 g of each AMVA emulsion (AMVA-1, AMVA-2, AMVA-3 and AMVA-4) was precisely dispensed into separate 100 mL centrifuge tubes, followed by the addition of 4 g bentonite to each. After thorough homogenization, the suspensions were centrifuged at 3000 rpm for 3 min, removed, and allowed to stand undisturbed for 2 h. Subsequent visual inspection revealed the extent of mud-ball agglomeration, thereby providing an effective index of the emulsions’ film-forming capacity and enabling the determination of the optimal AM content. The resulting morphologies of the bentonite pellets are illustrated in Figure 10.

Table 3 summarizes the influence of acrylamide (AM) content on the moldability of the film-forming agent. At an AM fraction of 3.7 wt%, the emulsion appears as a bluish-white fluid and bentonite aggregates into coherent clumps after centrifugation. At 1 wt% AM, the sodium bentonite pellets remain dispersed following soaking and centrifugation. When the AM content is increased to 7 wt%, pellet aggregation is observed; however, the corresponding emulsion exhibits compromised fluidity. At 10 wt% AM, the emulsion becomes highly viscous and the pellets are loosely packed and poorly consolidated. Consequently, 3.7 wt% AM (relative to total monomer) represents the optimal formulation under the tested conditions. This behavior is attributed to the amide groups (–CONH_2_) of AM, which can form hydrogen bonds with surface hydroxyl groups on the bentonite particles. These interactions enhance interfacial adhesion, resulting in the formation of a uniform and stable film that effectively prevents water from increasing into the pellets.

#### 2.5.2. Effect of Molar Ratio of VAc to AM on Film-Forming Properties of AMVA Emulsions

**Figure 11:**
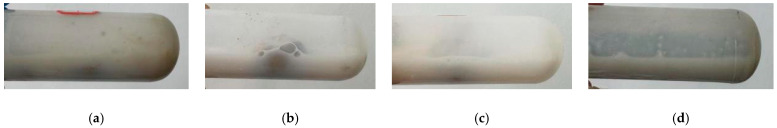
Effect of molar ratio of VAc to AM on the film-forming properties of emulsions; (**a**) AMVA-5 (AM:VAc = 1:2); (**b**) AMVA-6 (AM:VAc = 1:3); (**c**) AMVA-2 (AM:VAc = 1:4); (**d**) AMVA-7 (AM:VAc = 1:5).

40 g of each AMVA emulsions (AMVA-5, AMVA-6, AMVA-2, and AMVA-7) were subjected to film-forming assessments. The sedimentation behavior of bentonite was monitored to quantify the influence of VAc content on the emulsions’ film-forming capacity and to determine its optimal proportion. Figure 11 illustrates the impact of varying AM:VAc molar ratios on the stability of bentonite pellets, thereby corroborating the role of VAc in film formation and facilitating identification of the optimal ratio.

As indicated in Figure 11 and Table 4, the VAc content exerts a pronounced influence on emulsion stability. At AM:VAc molar ratios of 1:2 and 1:5, the emulsions appear as faint, milky dispersions lacking evident Tyndall scattering; the corresponding bentonite–emulsion mixtures form fragile clusters that redisperse upon agitation, indicating limited stability. Conversely, ratios of 1:3 and 1:4 yield emulsions exhibiting a distinct bluish opalescence. Although AMVA-6 (1:3) and AMVA-2 (1:4) both produce cohesive pellets after centrifugation, the aggregates generated by AMVA-6 display a smoother, more compact surface morphology, reflecting superior inter-particle consolidation. The bentonite aggregation assay further corroborates this enhancement, revealing a higher aggregation efficiency and thus improved stability. Collectively, these findings establish an optimal AM:VAc molar ratio of 1:3.

#### 2.5.3. Effect of Cellulose Content on Emulsion Film-Forming Properties

**Figure 12:**
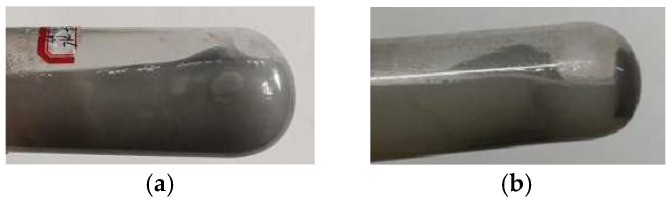
Impact of cellulose content on the film-forming properties of emulsions; (**a**) AMVA-8 (1 wt% cellulose content); (**b**) AMVA-9 (1.6 wt% cellulose content).

To ascertain the optimal cellulose content within AMVA emulsions, systematic film-forming evaluations were conducted on AMVA-8 and AMVA-9. Figure 12 illustrates the influence of varying cellulose contents on the emulsions’ film-forming performance.

Table 5 evidences that cellulose content exerts a decisive influence on emulsion stability. At cellulose content of 1.0 wt% and 1.6 wt%, the emulsions exhibit a pale, milky color and poor appearance. Upon admixture with bentonite and subsequent centrifugation, however, the resulting dispersions demonstrate markedly enhanced stability.

Among the bentonite pellets examined, those immersed in the AMVA formulation containing 1.6 wt% cellulose exhibited the most pronounced agglomeration (Figure 12b); the pellets remained intact and resisted dispersion. This behavior is attributed to the cellulose chains forming a three-dimensional, cross-linked network with reactive moieties—particularly acrylamide—within the inhibitive solution. The resulting supramolecular scaffold significantly enhances the mechanical integrity and colloidal stability of the interfacial film enveloping the bentonite particles, thereby markedly improving their clumping efficacy.

## 3. Materials and Methods

### 3.1. Materials

Allyloxynonylphenol sulfonate (DNS), Sodium dodecyl benzene sulfonate (SDBS) were obtained from Shanghai Jadel Chemical Technology Co., Ltd. (Shanghai, China); Alkylphenol ethoxylates (OP-10), Acrylamide (AM) and Potassium peroxydisulfate (KPS) were produced by Tianjin Kemiou Chemical Reagent Co., Ltd. (Tianjin, China); Sodium dodecyl benzene sulfonate was purchased from Tianjin Bodi Chemicals Co., Ltd. (Tianjin, China); γ-Methacryloxypropyltrimethoxysilane (γ-MPS) was supplied by Wuhan Xinyang Ruihe Chemical Technology Co., Ltd. (Wuhan, China); Methyl methacrylate (MMA), Vinyl acetate (VAc) and Styrene (St) were produced by Shandong Keyuan Biochemical Co., Ltd. (Jinan, China); and Sodium-based bentonite (Na-BT) was obtained from Tianjin Huasheng Chemical Reagents Co., Ltd. (Tianjin, China).

### 3.2. Methods

An AMVA emulsion was synthesized via semi-continuous emulsion polymerization at 80 °C. DNS, OP-10, and SDBS were first dissolved in deionized water under agitation. One-half of the aqueous acrylamide solution was added to the surfactant mixture. Subsequently, one-fourth of the monomer mixture (MMA, γ-MPS, VAc, and St) was introduced dropwise via a dropping funnel, followed by the addition of one-fourth of the initiator after 10 min of reaction. The remaining acrylamide solution, monomers, and initiator were then fed continuously over 1 h at 80 °C. Upon completion, a bluish-white AMVA emulsion was obtained.

**Figure 13:**
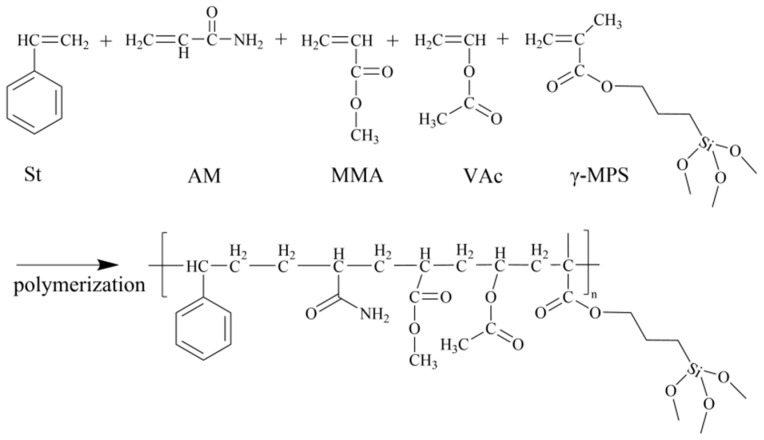
Synthesis mechanism of AMVA emulsion.

The influence of the acrylamide (AM)–vinyl acetate (VAc) ratio on inhibitor performance was systematically investigated. AMVA-1, AMVA-2, AMVA-3, and AMVA-4 were synthesized with fixed AM:VAc = 1:4 and AM mass fractions of 1.0 wt%, 3.7 wt%, 7 wt%, and 10 wt%, respectively. AMVA-5, AMVA-6, and AMVA-7 were prepared at AM:VAc molar ratios of 1:2, 1:3, and 1:5, respectively, while maintaining an AM content of 3.7%. Detailed formulations are provided in Table 6, and the proposed polymerization mechanism is depicted in Figure 13.

### 3.3. Mechanism Analysis

#### 3.3.1. Particle Size Testing

The particle size of the dispersions was determined by dynamic light scattering (DLS) using a Malvern Zetasizer Nano ZS (Malvern Instruments, Great Malvern, UK). All AMVA emulsions were diluted with deionized water to the concentration recommended by the manufacturer. The Z-average diameter (Dz) served as the descriptor of mean particle size, while the polydispersity index (PDI) quantified the breadth of the distribution. All relevant parameters were automatically computed by the instrument’s integrated software (Malvern Zetasizer Software, version 7.1.3).

#### 3.3.2. Microscopic Analysis

The polarized optical microscopy (POM) was used to observe the morphology of the AMVA emulsion. In the experiment, a DM3000 polarized optical microscope was used (Leica Microsystem, Wetzlar, Germany), and the samples to be tested were uniformly coated on the surface of slides, and the film thickness was controlled within the range of 100 μm, and the samples were observed and analyzed at the magnification of 20× of the objective lens.

#### 3.3.3. FTIR

The AMVA emulsion was purified, dried, and ground into a powder. The molecular structure of the product was analyzed using an IRPrestige-21 Fourier transform infrared (FTIR) spectrometer (Shimadzu, Tokyo, Japan). Spectra were recorded at room temperature over a wavenumber range of 4000~400 cm^−1^.

#### 3.3.4. TG Analysis

The decomposition temperature of the AMVA emulsion samples was analyzed using a Q500 thermogravimetric analyzer (TA Instruments, New Castle, DE, USA). The measurements were conducted under a nitrogen atmosphere with a heating rate of 10 °C/min. The thermal degradation was tested over a temperature range of 40–600 °C. Each sample was placed in a platinum crucible, and a single test was performed for each.

#### 3.3.5. Film-Forming Ability Test

At 25 °C, 4 g of sodium bentonite and 40 g of the inhibitor were weighed and placed into a centrifuge tube. After thorough mixing, the tube was placed in a centrifuge operated at 3000 rpm for 3 min. Upon completion of centrifugation, the samples were left undisturbed for 2 h to observe the clumping of sodium bentonite pellets. This phenomenon was used to characterize the film-forming properties of the AMVA emulsion and to determine the optimal monomer ratio. The underlying mechanism is that the AMVA emulsion forms a thin film on the surface of sodium bentonite pellets, which encapsulates them and prevents moisture penetration, thereby inhibiting their dispersion.

## 4. Conclusions and Recommendations

In this study, an acrylamide-based AMVA emulsion (AM–VAc–MMA) was synthesized via emulsion polymerization for deployment in water-based drilling fluids to enhance wellbore stability.

(1) By systematically varying the acrylamide (AM) and cellulose contents as well as the vinyl acetate (VAc):AM molar ratio, and evaluating the resulting formulations via moldability assays, the optimal composition and film-forming efficacy of the AM–VAc–cellulose emulsion were determined. Centrifugation tests revealed that an AM mass fraction of 3.7 wt% yielded non-dispersed bentonite agglomerates, indicative of superior film formation. Thermogravimetric analysis confirmed that AMVA-2 exhibited the highest thermal stability under these conditions. Furthermore, a VAc:AM molar ratio of 1:3 and a cellulose content of 1.6 wt% were identified as critical parameters for maximizing emulsion stability and film integrity.

(2) By dynamic light scattering and optical microscopy, particle size analysis and microscopy revealed that AM–VAc–cellulose emulsions exhibit mean diameters of approximately 40~120 nm, with a narrow, monodisperse distribution observed under the optimized formulation.

(3) The results of this study indicate that the synthesized AMVA emulsion has excellent emulsion stability and film-forming ability. The film-forming performance tests further confirm its effectiveness as a bentonite inhibitor. However, we must also acknowledge that the analytical approach used in this study has certain limitations. When observing the microstructure of the emulsion under a microscope, contrast staining represents a feasible and effective improvement. Future research will focus on overcoming this limitation.

## Figures and Tables

**Table 1:** Different cellulose proportions in AMVA emulsions.

Sample Name	AM/wt%	MMA/wt%	VAc/wt%	Cellulose/wt%	γ-MPS/wt%	St/wt%
AMVA-8	3.7	55.6	14.8	1	7.4	18.5
AMVA-9	3.7	55.6	14.8	1.6	7.4	18.5

**Table 2:** Thermogravimetric data for AMVA emulsion.

Sample Name	T_Initial_/°C	T_1/2_/°C	T_max_/°C
AMVA-1	117	379	415
AMVA-2	246	386	392
AMVA-3	160	385	380
AMVA-4	120	261	229

**Table 3:** Effect of AM content on inhibitor moldability.

Sample Name	*w*(AM)/%	Product Stability	Bentonite Agglomeration Test
AMVA-1	1 wt%	Milky white colloidal	Poor clumping after centrifugation
AMVA-2	3.7 wt%	Milky white liquid with bluish color	Clumping after centrifugation
AMVA-3	7 wt%	Milky white consistency, large amount of precipitation	Clumping after centrifugation
AMVA-4	10 wt%	Milky white consistency, large amount of precipitation	Poor clumping after centrifugation

**Table 4:** Effect of molar ratio of VAc to AM on inhibitor moldability.

Sample Name	*n*(VAc):*n*(AM)	Product Stability	Bentonite Agglomeration Test
AMVA-5	1:2	Milky white color is lighter, blue light is not obvious	Clusters after centrifugation, dispersed after shaking
AMVA-6	1:3	bluish milky white emulsion	Clumps after centrifugation, does not disperse when shaken
AMVA-2	1:4	Milky white color is lighter, blue light is not obvious	Clumps after centrifugation, does not disperse when shaken
AMVA-7	1:5	Milky white color is lighter, blue light is not obvious	Clusters after centrifugation, dispersed after shaking

**Table 5:** Effect of cellulose content on film-forming property.

Sample Name	Fiber Content/wt%	Product Stability	Bentonite Agglomeration Test
AMVA-8	1.0	Lighter milky white	Centrifuged and clumped
AMVA-9	1.6	Lighter milky white	Centrifuged and clumped

**Table 6:** Composition of different formulations of AMVA emulsion.

Sample Name	AM/(wt%)	MMA/(wt%)	VAc/(wt%)	γ-MPS/(wt%)	St/(wt%)
AMVA-1	1.0	55.6	14.8	7.4	18.5
AMVA-2	3.7	55.6	14.8	7.4	18.5
AMVA-3	7.0	55.6	14.8	7.4	18.5
AMVA-4	10.0	55.6	14.8	7.4	18.5
AMVA-5	3.7	55.6	7.4	7.4	18.5
AMVA-6	3.7	55.6	11.1	7.4	18.5
AMVA-7	3.7	55.6	18.5	7.4	18.5

## Data Availability

All data generated or analyzed during this study are included in this article. Further inquiries can be directed to the corresponding author.
